# *KRAS* and *BRAF* Mutation Detection: Is Immunohistochemistry a Possible Alternative to Molecular Biology in Colorectal Cancer?

**DOI:** 10.1155/2015/753903

**Published:** 2015-04-23

**Authors:** Nicolas Piton, Francesco Borrini, Antonio Bolognese, Aude Lamy, Jean-Christophe Sabourin

**Affiliations:** ^1^Laboratory of Cancer Genetics, Department of Pathology, Rouen University Hospital, 76031 Rouen, France; ^2^Department of Surgery “Pietro Valdoni”, Rome University Hospital, La Sapienza, 00161 Rome, Italy

## Abstract

*KRAS* genotyping is mandatory in metastatic colorectal cancer treatment prior to undertaking antiepidermal growth factor receptor (EGFR) monoclonal antibody therapy. *BRAF* V600E mutation is often present in colorectal carcinoma with CpG island methylator phenotype and microsatellite instability. Currently, *KRAS* and *BRAF* evaluation is based on molecular biology techniques such as SNaPshot or Sanger sequencing. As molecular testing is performed on formalin-fixed paraffin-embedded (FFPE) samples, immunodetection would appear to be an attractive alternative for detecting mutations. Thus, our objective was to assess the validity of KRAS and BRAF immunodetection of mutations compared with the genotyping reference method in colorectal adenocarcinoma. *KRAS* and *BRAF* genotyping was assessed by SNaPshot. A rabbit anti-human KRAS polyclonal antibody was tested on 33 FFPE colorectal tumor samples with known *KRAS* status. Additionally, a mouse anti-human BRAF monoclonal antibody was tested on 30 FFPE tumor samples with known *BRAF* status. KRAS immunostaining demonstrated both poor sensitivity (27%) and specificity (64%) in detecting *KRAS* mutation. Conversely, BRAF immunohistochemistry showed perfect sensitivity (100%) and specificity (100%) in detecting V600E mutation. Although molecular biology remains the reference method for detecting *KRAS* mutation, immunohistochemistry could be an attractive method for detecting *BRAF* V600E mutation in colorectal cancer.

## 1. Introduction

Colorectal cancer is the second most common cause of cancer death in the Western world, and its incidence is increasing [1]. Approximately 50% of colorectal cancer patients eventually develop metastatic disease, for which systemic palliative treatments are usually administered.

Treatment options for patients with metastatic colorectal cancer (mCRC) have changed considerably in recent years with the introduction of antiepidermal growth factor receptor (EGFR) therapies targeting EGFR transduction cascade, which is one of the leading oncogenic pathways used by tumoral cells.

The Food and Drug Administration (FDA) and the European Medicines Agency (EMEA) have approved the use of anti-EGFR monoclonal antibodies, cetuximab and panitumumab, in patients with mCRC without* KRAS* (Kirsten rat sarcoma viral oncogen homolog) mutation. The KRAS protein, belonging to the large superfamily of guanine guanosine-50-triphosphate (GTP) and guanine guanosine-50-diphosphate (GDP) binding proteins, is a powerful downstream effector in the EGFR transduction cascade. Somatic* KRAS* mutations are detected in about 40% of colorectal cancer and lead to an abnormal affinity of KRAS for GTP with permanent activation of the transduction cascade.* KRAS* mutations have been identified as a reliably strong negative predictive factor to anti-EGFR monoclonal antibody therapies in mCRC patients [2–4].* KRAS* mutation screening has been mandatory in Europe since July 2008 and aims to restrict treatment of mCRC to cetuximab and panitumumab in patients with wild-type* KRAS* tumors [5, 6].

*BRAF* (v-Raf murine sarcoma viral oncogene homolog B1) is a member of the* RAS/RAF* family, encoding a serine-threonine protein kinase that is involved in the MAPK (mitogen-activated protein kinase) signaling cascade.* BRAF* acts as a direct effector of RAS and promotes tumor growth and survival through the activation of MEK (*MAPK/*ERK kinase) [7]. Most frequent activating mutations of* BRAF* found in colorectal cancer almost invariably result in valine substituting glutamate at residue 600 (BRAF V600E) and occur at a frequency of 10–15% [8]. In colorectal cancer,* BRAF* mutations are tightly correlated with two molecular features:  CpG island methylator phenotype (CIMP) and microsatellite instability (MSI). Approximately 11–76% of MSI tumors harbor a* BRAF* mutation versus only 0–15% of microsatellite stable (MSS) tumors [7].* BRAF* mutations are absent in Lynch syndrome, with defective germline mutation in mismatch repair (MMR) system, but usually present in sporadic MSI colorectal tumors. As a consequence,* BRAF* V600E analysis is used to differentiate sporadic MSI colorectal cancers from Lynch syndrome cases [9].

*KRAS* and* BRAF* status is evaluated using molecular technologies after genomic tumoral DNA extraction such as Sanger sequencing, SNaPshot, real-time PCR, or other validated methods [10]. Despite molecular pathology being the “natural evolution” of anatomical pathology, not all pathology departments are currently equipped to perform such analyses. Furthermore, financial issues due to the high cost of equipment and reagents for molecular pathology could also lead to a switch to an alternative method of detection. Since molecular testing is mainly performed on formalin-fixed paraffin-embedded (FFPE) tumor samples, which are stored in the pathology department, immunodetection appears to be an attractive alternative for such mutation detection. Indeed, immunohistochemistry is an approved method for discriminating which patients can benefit from specific cancer therapy, such as determination of HER2 expression in breast or gastric cancer. Moreover, the pathologist could also perform* KRAS* and* BRAF* mutation screening by immunohistochemistry at the same time as histopathology diagnosis, resulting in considerable saving of time.

In order to validate this new approach, we evaluated a rabbit anti-human KRAS polyclonal antibody, directed against an internal region of human KRAS protein, and a mouse anti-human BRAF monoclonal antibody. Then, we correlated the results with mutation status obtained by DNA sequence analysis in human colorectal carcinoma samples.

## 2. Materials and Methods

### 2.1. Molecular Analyses

Formalin-fixed paraffin-embedded tumor specimens were obtained from Sapienza University Hospital (Rome, Italy) and Rouen University Hospital (Rouen, France). A total of 63 samples were randomly selected from metastatic colorectal cancer patients referred to our laboratories between May 2009 and October 2012 for* KRAS* genotyping.

Serial sections of primary colorectal tumors were cut from each paraffin block and placed on glass slides: one 4 *µ*m thick section was stained with hematoxylin & eosin (H&E) for histopathological examination and another 4 *µ*m thick section was used for immunohistochemical techniques. The following five 10 *µ*m thick sections were processed for tumor DNA preparation. The microtome razor blade was changed between each FFPE tumor sample and the paraffin sections were processed individually to avoid cross-contamination.

H&E preparation enabled tumor area delimitation and visual estimation of tumor cell percentage. To minimize nonmalignant tissue and stromal contaminating inflammatory cells, tumor areas, previously highlighted by a pathologist on H&E preparation, were macrodissected on each of the five 10 *µ*m thick sections placed on glass slides using a single-use sterilized scalpel.

*KRAS* and* BRAF* genotyping was performed according to protocols as previously reported [2, 11]. Briefly, for SNaPshot analysis, after genomic DNA extraction using the “RecoverAll Total Nucleic Acid Isolation Kit for FFPE Tissues” (Applied Biosystems) as recommended by the supplier,* KRAS* exon 2 (codons 12 and 13) and* BRAF* exon 15 were amplified by PCR (Polymerase Chain Reaction) using specific primers. SNaPshot primer extension reaction was performed including ddNTPs (dideoxynucleotide triphosphate) labeled with fluorochromes, specific SNaPshot primers, and 2 *µ*L of purified PCR products. After migration in an automated sequencer (ABI PRISM 3130xl) the results were analyzed using GeneMapper software version 4.0 (Applied Biosystems). SNaPshot multiplex amplification products of* KRAS* and* BRAF* allowed visualization of any mutations arising in these specific positions: c.34G, c.34G, c.35G, c.37G, c.38G (for* KRAS*), and c.1799T (for* BRAF*).

### 2.2. Sample Selection

For the KRAS study, the population sample was composed of 33 patients including 11 cases with cancers harboring the most frequent* KRAS* mutations (G12A, G12C, G12D, G12V, G13D, G12S, and G12R) without mutation of* BRAF* and 22 cases with* KRAS* and* BRAF* wild-type status. For the BRAF study, we selected 30 patients including 20 patients with a tumor harboring a c.1799T>A (V600E)* BRAF* mutation without any* KRAS* mutation (10 MSI tumors and 10 MSS tumors) and 10 patients presenting with MSS tumor without* BRAF* or* KRAS* mutations.

### 2.3. Immunohistochemical Analysis

Immunohistochemical staining was performed on 4 *μ*m sections of FFPE blocks. Deparaffinization and rehydration were performed with xylene and alcohol. After washing with distilled water, sections were preheated in a microwave oven for 30 minutes at 99°C at a 10 mmol/L concentration of citrate buffer (pH 6). After rinsing in water, endogenous peroxidase activity was blocked by immersion in 3% hydrogen peroxide for 5 minutes. Then, sections were incubated for 1 hour at room temperature with a rabbit polyclonal antibody against KRAS (Rabbit Anti-Human k-Ras Polyclonal Antibody, dilution 1 : 100; Spring Bioscience, California) or for 16 minutes at 37°C with a mouse monoclonal antibody against BRAF V600E protein (VE1 clone; Spring Bioscience, California).

Immunoreactivity was revealed with the DAKO EnVision system using 3,3′-diaminobenzidine tetrahydrochloride (DAKO) chromogen with hematoxylin counterstaining. Negative control staining was performed by omitting the primary antibody.

Optimization of each antibody included samples of FFPE prostate cancer tissue as indicated by the supplier and two lines of colorectal cancer cells, namely, HCT116 and HT29, which harbor, respectively,* KRAS* mutation and* BRAF* V600E mutation.

Each immunostained slide was examined and scored independently by two pathologists (Jean-Christophe Sabourin and Francesco Borrini) without prior knowledge of any clinicopathological or molecular data. All sections for which the two observers disagreed were reevaluated and, after discussion, final agreement was achieved.

Immunoreactivity was scored by taking into account the percentage of positive tumor cells with no relation to signal intensity: tumors were assessed as KRAS negative or BRAF negative if 90% of the cancer cells were unstained (<10% of positive cells) and as KRAS positive or BRAF positive if more than 10% of cells were immunostained.

### 2.4. Statistical Analysis

Mutation status by molecular biology was considered as the gold standard technique for mutation detection. For KRAS and BRAF immunostainings, sensitivity and specificity were calculated using a contingency table comprising positive cases (true or false) and negative cases (true or false).

## 3. Results

**Figure 1:**
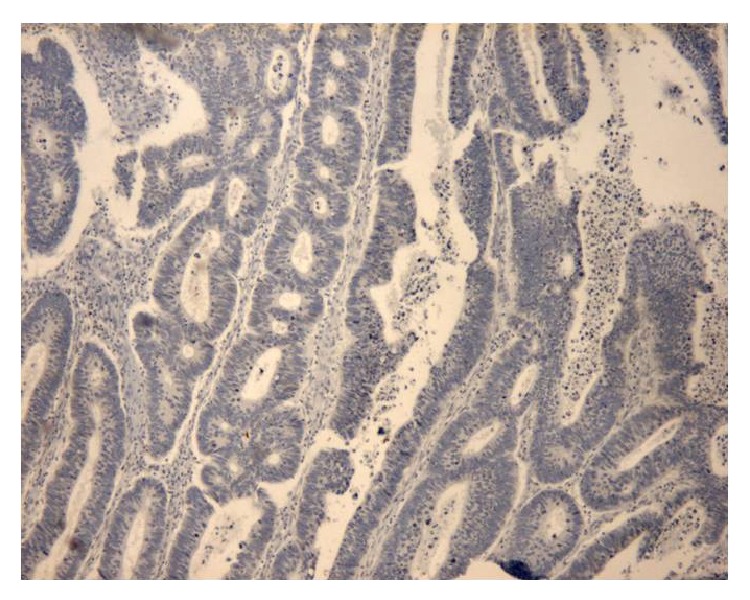
Immunohistochemical analysis of* KRAS* mutated colorectal cancer with absence of staining, ×50 HPF.

**Figure 2:**
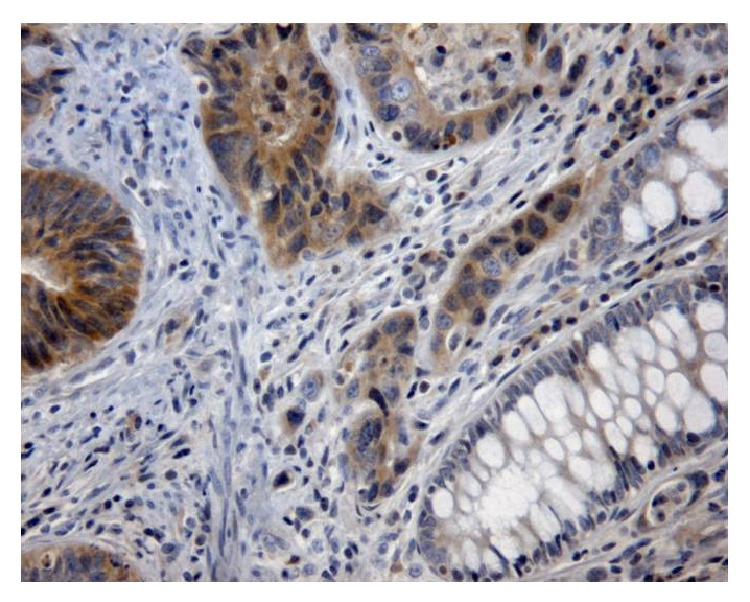
Immunohistochemical analysis of* KRAS* mutated colorectal cancer with positive staining, ×200 HPF.

**Figure 3:**
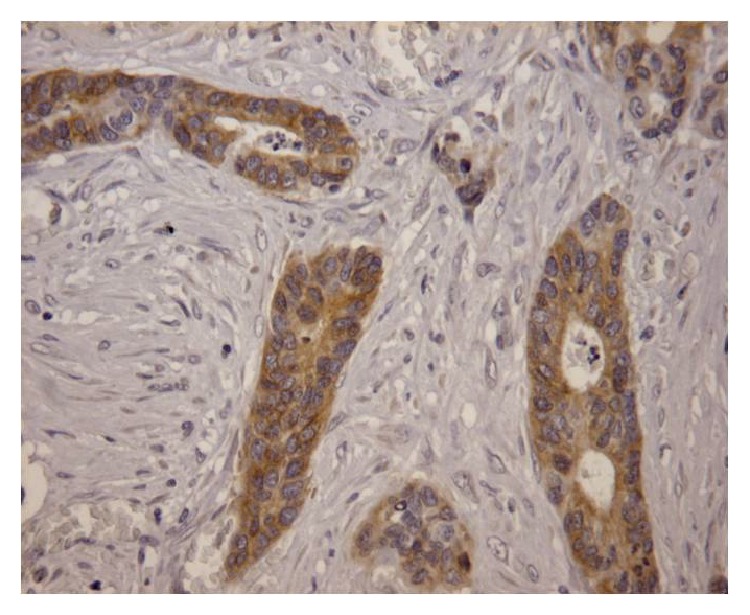
Immunohistochemical analysis of* KRAS* wild-type colorectal cancer with positive staining, ×200 HPF.

The KRAS study enrolled 11 patients with tumoral CRC* KRAS* mutation status and 22 patients with wild-type* KRAS* status. Patient characteristics, tumoral mutation data, and staining status are listed in Table 1. Mean age was 63 years (standard deviation = 10). There were 23 male patients with a mean age of 64 years (standard deviation = 10) and 10 female patients with a mean age of 61 years (standard deviation = 9). When present, KRAS protein expression was principally located in the cytoplasm, displaying a granular pattern, and only occasionally in cytoplasm membrane (Figures 1, 2, and 3). Distinct KRAS expression in neoplastic areas was observed in 11 (33%) patients; in the remaining 22 cases, no KRAS expression was observed. The sensitivity of immunostaining in detecting a* KRAS* mutation was calculated at 27%, whereas the specificity of this method was calculated at 64% (Table 2).

**Figure 4:**
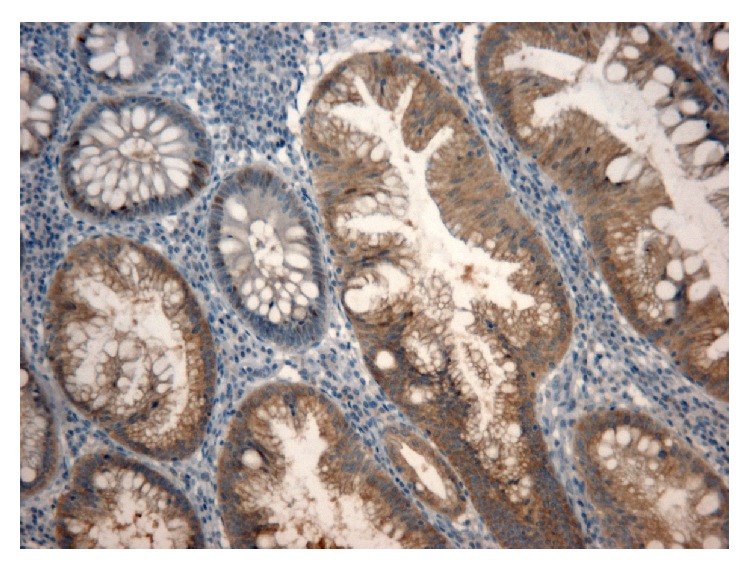
Expression of BRAF V600E in area of hyperplasia contiguous to* BRAF* mutated cancer, with absence of staining in normal mucosae ×50, 100 HPF.

**Figure 5:**
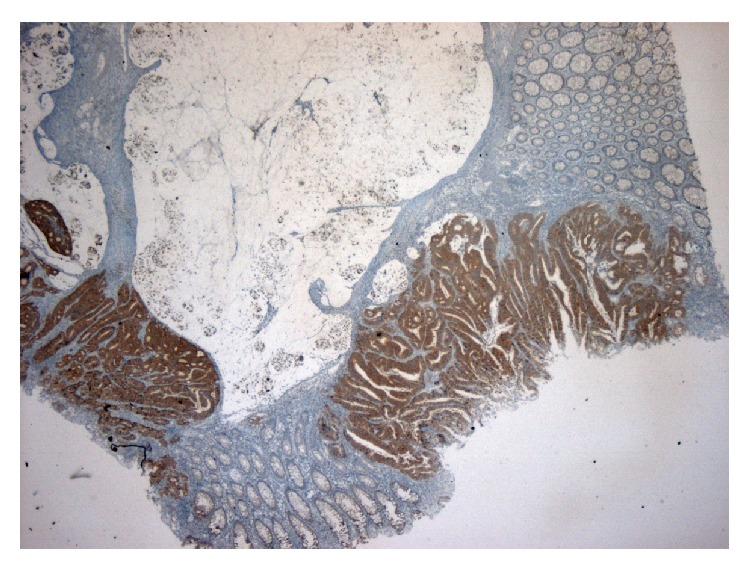
Immunohistochemical analysis of* BRAF* mutated colorectal cancer with positive staining, ×50 HPF.

The BRAF study enrolled 20 patients with tumoral CRC* BRAF* mutation (comprising 10 patients with MSI tumor and 10 patients with MSS tumor) and 10 patients with tumoral CRC* BRAF* wild type and MSS. Patient characteristics, tumoral mutation data, and staining status are listed in Table 3. The mean age of the sample population was 60 years (standard deviation = 12). There were 19 male patients with a mean age of 61 years (standard deviation = 13) and 11 female patients with a mean age of 59 years (standard deviation = 10). When positive, BRAF staining was homogenous, marked, or moderate. Distinct BRAF expression in neoplastic areas was observed in 20 (66%) patients; in the remaining 10 cases, no BRAF expression was observed. As expected, BRAF protein expression was located in the cytoplasm, with finely granular cytoplasmic staining (Figures 4 and 5). Any nuclear staining was ignored and not scored. Interestingly, one of the* BRAF* mutated cancers was contiguous to a hyperplastic/serrated area without areas of dysplasia. In this area, we observed decreased intensity of cytoplasmic staining compared to the invasive component of the tumor (Figures 4 and 5). By subgrouping these patients according to their tumoral* BRAF* genotyping, we found that BRAF expression was present in the 20 mutated patients, independently of microsatellite status, and was absent in the 10 nonmutated cases. Thus, both sensitivity and specificity of this technique were 100% (Table 4).

## 4. Discussion

We are aware that molecular pathology is the natural evolution of anatomical pathology. In contrast with immunohistochemistry which has not radically changed pathology processing, molecular pathology requires more fundamental technical adjustments and more expensive laboratory equipment.

The economic implications of customizing anti-EGFR therapy based on* KRAS* status were recently evaluated by Shankaran et al. using estimated incidence rates for new mCRC cases diagnosed in the United States. Based on an annual incidence of 29,762 new cases of mCRC, the cost of upfront* KRAS* molecular testing was calculated at $13 million (i.e., $452 per patient) [12]. An immunohistochemical approach is less expensive than molecular pathology, with an average cost of $50 per antibody tested [13].

This immunohistochemical approach, which is a valid resource for identification of tumoral targets accessible for therapy (i.e., CD117 in gastrointestinal stromal tumors [14] or HER2 in breast and gastric cancer [15, 16]), has not yet found an application in colorectal cancer. Screening mCRC with anti-EGFR antibodies has rapidly shown its limits in selecting patients for anti-EGFR therapies [17]. In mCRC, the only predictive marker (a negative marker of response to those treatments) is the determination of somatic* RAS* mutation. Thus, morphological detection using immunohistochemistry appears to be an attractive alternative to molecular screening. Indeed, we postulated that the* KRAS* mutations occurring in codons 12 and 13 of exon 2, by inducing a stabilization of the protein in a constitutive activation state, could also allow detection of this protein by immunohistochemistry. By using an antibody directed at KRAS (but at a nonmutated protein), we expected to visualize mutated KRAS protein in tumor cells of mutated carcinomas (or at least to detect an “overexpression” in mutated cases). Our data were unable to support this hypothesis.

Recent publications have reported the possibility of immunodetection of mutated protein as EGFR [18, 19] or BRAF [20]. Yu et al. recently generated monoclonal antibodies specific to the more frequently mutated EGFR protein (exon 19 E746-A750 deletions and exon 21 L858R mutation) [21]. They reported a 92% sensitivity of immunohistochemical assays in 340 non-small-cell carcinomas of lung specimens compared to 99% for classical DNA sequencing. The same results were found using a specific monoclonal antibody directed against V600E* BRAF* mutation [20] which only reacts with the protein product of the V600E mutant and not with protein associated with other mutations of* BRAF* [22]. In this context, we decided to evaluate the utility of BRAF V600E immunohistochemistry. The complete concordance observed in our study is comparable to published data [23–25].

These results strongly argue in favor of an immunohistochemical approach to exclude Lynch syndrome in microsatellite high (MSI-H) colorectal cancers, as suggested in a very recent publication [25].* BRAF* V600E mutation, which is virtually absent in hereditary colorectal cancer, is often present in sporadic CRC that has a CpG island hypermethylation phenotype (CIMP-high), resulting in hypermethylation of promoter regions.* BRAF* mutated CIMP-high CRC is frequently MSI-H, as the* MLH1* promoter has been methylated, resulting in an MLH1-deficient tumor [26].

Immunodetection of* BRAF* V600E mutation could also provide prognostic information. Recent data on adjuvant studies in patients with stage II/III colon cancer [27] and in metastatic disease [28] indicate that* BRAF* V600E mutation is associated with worse clinical outcome. The predictive role of* BRAF* mutation in colorectal cancer is currently debated, but a recent meta-analysis indicated that* BRAF* mutation is a predictive biomarker of poor prognosis in mCRC patients treated by anti-EGFR monoclonal antibodies, especially in* KRAS* wild-type patients [29].

In an era of personalized medicine for* BRAF* mutation in metastatic melanomas, a targeted therapy has been investigated in advanced colorectal cancer harboring a mutation of* BRAF*. The results of* BRAF* V600E inhibition in CRC are disappointing, compared to expectations raised in preclinical models and case reports [30, 31].

Morphological control of histological sections stained by immunohistochemistry allows evaluation of tumor areas with different phenotypic expressions. Recent studies have given evidence that morphologically homogeneous tumors may actually have very different genotypic characteristics [32]. Many hopes were sustained by the idea that a single biopsy could allow tumor characterization according to molecular profile and thus effectively permit best treatment for the patient. However, this tumoral heterogeneity may be one cause of the failure of targeted therapeutics: the presence of a nonsensitive subclone leads to its selection by the treatment and then to the recurrence of the disease. Thus, in clinical oncology, it seems important to take into account this tumoral heterogeneity and so to develop new strategies for molecular characterization. Immunohistochemistry is a powerful tool and represents a valid morphological support for phenotype characterization and especially detection of heterogeneity within tumour cells. However, its validity relies on the specificity of antibodies which must recognize only the mutated protein.

Regarding time spent by the laboratory, immunohistochemistry can easily be performed in one working day, whereas molecular techniques require a minimum of two working days.

Immunohistochemical evaluation could also be useful in cases where the quality of the material is not suitable for molecular investigation: it is possible to assess diagnosis of malignant tumors only by the presence of scattered cells in the specimen. On the contrary, molecular techniques require at the very least 5% of tumoral cells in the tested sample.

To summarize, by using an antibody directed at KRAS (but not specific to mutated protein), we failed to distinguish* KRAS* mutated tumors from* KRAS* wild-type tumors in mCRC. Thus, development of a monoclonal antibody designed against exon 2 codons 12 and 13 mutated* KRAS* domain (which represent more than 90% of KRAS mutations) could ultimately facilitate the screening of mCRC patients for anti-EGFR therapies. Indeed, our data support the use of immunohistochemistry in detecting* BRAF* V600E mutation protein as an alternative to molecular testing in colorectal cancer. Immunohistochemistry could become a promising tool in prognostic and therapeutic decisions in the very near future.

## Figures and Tables

**Table 1:** KRAS, phenotype, and genotype.

Case	Sex	Age (years)	KRAS staining	Molecular status (protein effect)
1	M	75	Positive	Mutated (G12D/G13D)
2	M	68	Positive	Mutated (G12A)
3	F	65	Positive	Mutated (G12C)
4	M	53	Negative	Mutated (G12D)
5	M	72	Negative	Mutated (G12V)
6	M	61	Negative	Mutated (G13D)
7	M	72	Negative	Mutated (G12S)
8	F	59	Negative	Mutated (G12R)
9	F	61	Negative	Mutated (G13D)
10	M	41	Negative	Mutated (G12A)
11	F	66	Negative	Mutated G12C)
12	M	69	Negative	Wild type
13	F	52	Negative	Wild type
14	M	38	Negative	Wild type
15	M	69	Negative	Wild type
16	M	68	Negative	Wild type
17	M	68	Negative	Wild type
18	M	55	Negative	Wild type
19	F	72	Negative	Wild type
20	M	59	Negative	Wild type
21	M	67	Negative	Wild type
22	M	62	Negative	Wild type
23	M	57	Negative	Wild type
24	M	79	Negative	Wild type
25	M	75	Negative	Wild type
26	M	63	Positive	Wild type
27	M	72	Positive	Wild type
28	F	58	Positive	Wild type
29	M	53	Positive	Wild type
30	F	60	Positive	Wild type
31	F	42	Positive	Wild type
32	M	67	Positive	Wild type
33	F	70	Positive	Wild type

F: female; M: male; MSI: microsatellite-instable; MSS: microsatellite-stable.

**Table 2:** Contingency table for determination of *KRAS *mutation status using immunostaining.

	*KRAS* mutation status		
	Wild type	Mutated	Total
KRAS immunohistochemistry status			
−	14	8	22
+	8	3	11
Total	**22**	**11**	**33**

Sensitivity = 3/11 = 27%.

Specificity = 14/22 = 64%.

**Table 3:** BRAF, phenotype, and genotype.

Case	Sex	Age (years)	BRAF staining	Molecular status
A	F	55	Positive	Mutated, MSI
B	M	64	Positive	Mutated, MSI
C	M	72	Positive	Mutated, MSI
D	M	75	Positive	Mutated, MSI
E	M	71	Positive	Mutated, MSI
F	F	61	Positive	Mutated, MSI
G	F	81	Positive	Mutated, MSI
H	M	81	Positive	Mutated, MSI
I	M	65	Positive	Mutated, MSI
J	M	74	Positive	Mutated, MSI
K	M	54	Positive	Mutated, MSS
L	M	58	Positive	Mutated, MSS
M	M	23	Positive	Mutated, MSS
N	F	50	Positive	Mutated, MSS
O	M	50	Positive	Mutated, MSS
P	F	50	Positive	Mutated, MSS
Q	M	61	Positive	Mutated, MSS
R	M	62	Positive	Mutated, MSS
S	F	67	Positive	Mutated, MSS
T	M	56	Positive	Mutated, MSS
U	F	56	Negative	Wild type, MSS
V	M	63	Negative	Wild type, MSS
W	F	62	Negative	Wild type, MSS
X	F	42	Negative	Wild type, MSS
Y	M	56	Negative	Wild type, MSS
Z	F	64	Negative	Wild type, MSS
AA	F	61	Negative	Wild type, MSS
AB	M	60	Negative	Wild type, MSS
AC	M	52	Negative	Wild type, MSS
AD	M	54	Negative	Wild type, MSS

F: female; M: male; MSI: microsatellite-instable; MSS: microsatellite-stable.

**Table 4:** Contingency table for determination of *BRAF* mutation status using immunostaining.

	BRAF mutation status		
	wt	Mutated	Total
BRAF immunohistochemistry status			
−	10	0	10
+	0	20	20
Total	**10**	**20**	**30**

Sensitivity = 20/20 = 100%.

Specificity = 10/10 = 100%.
